# The impact of the size of bone substitute granules on macrophage and osteoblast behaviors in vitro

**DOI:** 10.1007/s00784-021-03804-z

**Published:** 2021-02-04

**Authors:** Masako Fujioka-Kobayashi, Hiroki Katagiri, Michihide Kono, Benoit Schaller, Tateyuki Iizuka, Ali-Farid Safi

**Affiliations:** 1 Department of Cranio-Maxillofacial Surgery, Inselspital, Bern University Hospital, University of Bern, Bern, Switzerland; 2 Department of Oral and Maxillofacial Surgery, School of Life Dentistry at Tokyo, The Nippon Dental University, Tokyo, Japan; 3 Advanced Research Center, School of Life Dentistry at Niigata, The Nippon Dental University, Niigata, Japan; 4 Department of Oral and Maxillofacial Surgery, Tokyo Medical University, Tokyo, Japan

**Keywords:** Bone substitutes, Granule size, Macrophages, Osteoblasts, Bone regeneration, In vitro

## Abstract

**Objective:**

Bone substitute (BS) size might influence the clinical outcomes of guided bone regeneration (GBR) procedures. The aim of the present study was to investigate the influence of BS size on macrophage (Mφ) and osteoblast behaviors in vitro.

**Materials and methods:**

Two different granule sizes (S and M/L) were assessed for four different commercial BSs: deproteinized bovine bone mineral (DBBM), biphasic calcium phosphate type 1 (BCP1), BCP type 2 (BCP2), and carbonate apatite (CO_3_Ap). The BSs were compared for their impacts on the cell viability and differentiation potential of THP-1-derived Mφs and human osteoblast-like Saos-2 cells.

**Results:**

The smaller granules showed higher material volumes and surface areas than the larger granules. Significantly higher viability of Mφs and Saos-2 cells was observed with the DBBM_L-size granules than with the DBBM_S-size granules. Gene expression experiments in Mφs revealed few differences between the two sizes of each BS, although higher CD206 mRNA levels were observed in the BCP1_L group and the CO_3_Ap_M group than in the respective S-size groups on day 1. Only DBBM showed significantly higher mRNA levels of osteogenic markers, including Runx2 and osteocalcin, in Saos-2 cells in the S-size group than in the L-size group.

**Conclusions:**

The S-size and L-size DBBM granules exhibited clear differences in cell outcomes: cells cultured on the S-size granules exhibited lower cell viability, higher osteopromotive ability, and no noticeable Mφ polarization changes.

**Clinical relevance:**

A smaller granule size might be advantageous due to greater bone regeneration potential in the use of DBBM granules to treat defects.

**Supplementary Information:**

The online version contains supplementary material available at 10.1007/s00784-021-03804-z.

## Introduction

The four essential functions of bone substitutes (BSs) are to promote osteogenesis, osteoinduction, osteoconduction, and osteointegration [1]. Apart from gold-standard autologous bone grafts, which possess these four elements, various BSs, including allografts, xenografts, and alloplasts, have been on the market and are recognized for their efficacy in bone regeneration [1, 2]. In particular, different types of BS granules are often and widely utilized for guided bone regeneration (GBR) in dentistry [2].

Deproteinized bovine bone mineral (DBBM) is one of the most widely utilized biomaterials in regenerative dentistry worldwide [3]. DBBM granules provide excellent osteoconductivity and promote the stability of new bone without causing adverse effects such as disease transmission [4, 5]. Furthermore, calcium phosphate-based synthetic BSs, such as biphasic calcium phosphate (BCP), which are composed of different concentrations of the stable phase, hydroxyapatite (HAp), and the more soluble phase, usually composed of β-tricalcium phosphate (β-TCP), have shown significant advantages due to their well-controlled bioactivity and balance between resorption and solubilization [6]. Another type of synthetic BS, carbonate apatite (CO_3_Ap), which contains 6–9 wt% carbonate in its apatitic structure (similar to that of natural bone), has also been recognized as an excellent osteopromotive BS, that is, interestingly, resorbed by osteoclasts [7–9].

Not only the material but also the size of a BS can affect bone regenerative potential. Interestingly, some studies have reported that the shape of the material influences tissue reactions in vivo [10–12]. Ghanaati et al. showed that differences in the size, shape, and porosity of β-TCP granules had an impact on material integration, multinucleated giant cell formation, and angiogenesis in a rat subcutaneous implant model [10]. Tanuma et al. reported that smaller octacalcium phosphate (OCP) granules combined with atelocollagen enhanced the regenerative potential of new bones compared to larger OCP granules in a rat critical-sized calvarial defect model [11]. Furthermore, Dawson et al. found that the increased porosity of HAp resulted in a significant increase in new bone formation due to the greater porosity and surface area of the recognized osteopromotive substrate in a rabbit femur defect model; there was more bone formation in porous Hap than in nonporous HAp in 80% of rabbits at 8 weeks and 100% of rabbits at 13 weeks [12].

It has been hypothesized that differences in granule size might influence biocompatibility as well as osteopromotive potential. Most commercially available BSs have been reported to have excellent biocompatibility and effectiveness in bone formation in vitro and in vivo. However, there are few reports that comprehensively show the effects of BS granule size on biocompatibility and osteopromotive potential; specifically, there are few in vitro data on the effects of different kinds of BS on macrophage (Mφ) polarization or osteoblast differentiation. Interestingly, it has been found that a population of tissue Mφs named “OsteoMacs” (osteal Mφs) is associated with immune cells in the bone microenvironment [13]. Furthermore, depletion of OsteoMacs in various in vivo settings led to a marked reduction in total bone mass [14]. Therefore, the cellular behavior of Mφs and osteoblasts cultured on BSs in vitro might reflect the osteopromotive potential of the BSs.

The aim of the present study was to investigate two different sizes (S size and M/L size) of four commercial BS granules, including DBBM (Bio-Oss® granules, Geistlich Pharma AG, Wolhusen, Switzerland), BCP1 (maxresorb® granules, botiss biomaterials GmbH, Zossen, Germany), BCP2 (Straumann® BoneCeramic™, Straumann Holding AG, Basel, Switzerland), and CO_3_Ap (Cytrans® granules, GC Corporation, Tokyo, Japan), to determine their effects on Mφs and osteoblasts. For each BS tested, the volume, surface area, and density were characterized by micro-CT analysis, and then each BS was further assessed for its effects on the viability and differentiation of Mφs derived from human monocyte THP-1 cells and human osteoblast-like Saos-2 cells.

## Materials and methods

### Materials

**Fig. 1:**
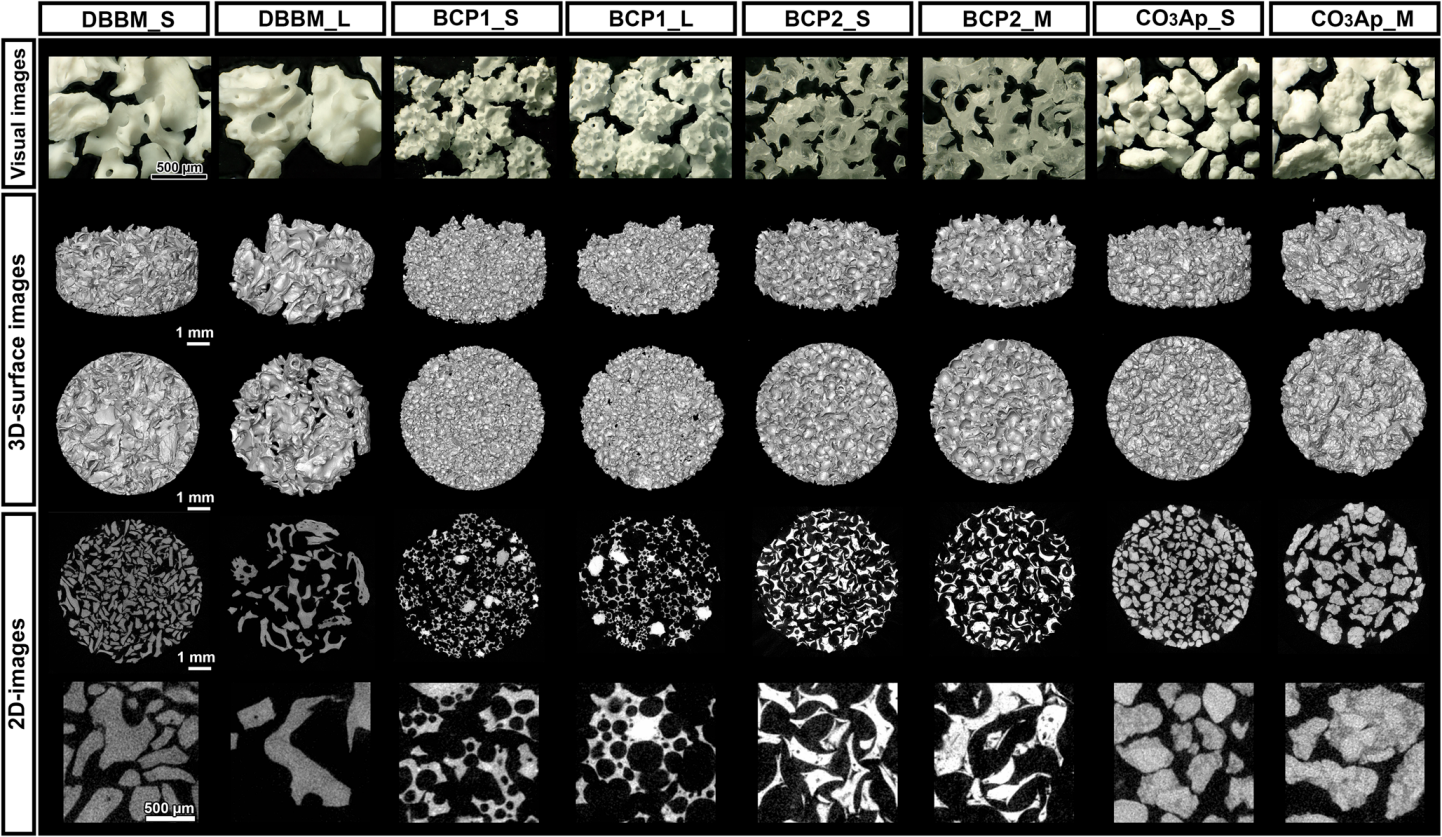
Visual images of the tested bone substitute (BS) materials and micro-CT views of the BS materials in a 6.4 mm diameter × 2 mm height cylinder

A list and images of the materials tested in the present study are shown in Table 1 and Fig. 1, respectively. Throughout the study assays, S-size and L-size granules were compared for DBBM and BCP1, and S-size and M-size granules were compared for BCP2 and CO_3_Ap. All BSs were aseptically prepared at the same size (φ6.4 mm in diameter × 2.0 mm in height) in 96-well plates or cell culture plate inserts (Transwell® inserts, Sigma, St. Louis, MO, USA) to standardize bulk volume, as previously reported [15].

**Table 1:** List of the tested bone substitutes

	Size	Product name	Manufacturer	Origin/components	Particle size (mm)
DBBM	S	Bio-Oss® granules	Geistlich Pharma AG, Wolhusen, Switzerland	Bovine bone	0.25–1.0
	L				1.0–2.0
BCP1	S	maxresorb® granules	Botiss biomaterials GmbH, Zossen, Germany	60% HAp, 40% β-TCP	0.5–1.0
	L				0.8–1.5
BCP2	S	Straumann® BoneCeramic™	Straumann Holding AG, Basel, Switzerland	60 % HAp, 40 % β-TCP	0.4–0.7
	M				0.5 – 1.0
CO_3_Ap	S	Cytrans® granules	GC Corporation, Tokyo, Japan	CO_3_Ap	0.3–0.6
	M				0.6–1.0

*HAp* hydroxyapatite, *β-TCP* beta-tricalcium phosphate, *CO*_*3*_*Ap* carbonate apatite

### Micro-CT analysis

The tested materials were subjected to micro-CT scanning using a desktop cone-beam scanner (microCT 40, Scanco Medical AG, Brüttisellen, Switzerland). The X-ray source was set at 70 kVp with 114 μA at a voxel size of 6 μm. The micro-CT images were then segmented, and the material volume, surface area, and density were calculated using 3D structural analysis software (Amira 6.1, Thermo Fisher Scientific Inc., Waltham, MA, USA).

### Cell culture

THP-1 human monocytes (ATCC, Manassas, VA, USA) and human osteoblast-like Saos-2 cells (Sigma) were used in the present study. The THP-1 cells were grown at 37 °C in a 5% CO_2_ atmosphere in RPMI 1640 supplemented with 10% fetal bovine serum, 100 units/mL penicillin, and 100 μg/mL streptomycin. To facilitate their differentiation into Mφs (M0_Mφs), THP-1 monocytes were treated with 100 ng/mL phorbol 12-myristate 13-acetate (PMA) for 24 h. The Saos-2 cells were cultured in a humidified atmosphere at 37 °C in growth medium consisting of McCoy’s 5A medium, 10% fetal bovine serum, and antibiotic-antimycotic serum (100 units/mL penicillin, 100 μg/mL streptomycin, and 250 ng/mL amphotericin B). The cells were detached from the plastic tissue culture plates using 0.25% EDTA-Trypsin prior to reaching confluency. For experiments lasting longer than 5 days, the medium was replaced twice weekly. Ethical approval was not necessary since commercial cell lines were used in the present study.

### Cell viability

Either M0_Mφs derived from THP-1 cells or Saos-2 cells were seeded on one of the BSs at a density of 5.0 × 10^4^ cells per well in 96-well culture plates. Luminescence cell viability assays (CellTiter-Glo®, Promega, Madison, WI, USA) were performed at 1 and 3 days after plating. Cell viability was quantified by a plate reader (TECAN Infinite200, Tecan Group Ltd., Männedorf, Switzerland).

### Real-time PCR analysis

Total RNA was harvested by an RNA Miniprep System (ReliaPrep™ RNA Cell Miniprep System, Promega, Madison, WI, USA), and cDNA was prepared using a reverse transcription system (GoScript™ Reverse Transcription System, Promega). Real-time PCR was performed on a real-time PCR machine (7500 Fast Machine, Applied Biosystems, Thermo) using SYBR Green Master Mix (GoTaq® qPCR Master Mix, Promega). The primers were generated with the primer sequences given in Supplemental Table 1. The ∆∆Ct method was used to calculate the gene expression levels after normalization according to the expression of glyceraldehyde 3-phosphate dehydrogenase (GAPDH). The data show the values relative to the mRNA levels in cells cultured on control tissue culture plastic.

### Undecalcified frozen section preparation and staining

M0_Mφs and Saos-2 cells were seeded on each BS at a density of 1.0 × 10^5^ cells per insert in 6.5-mm inserts^††^ with 0.4-μm pore polycarbonate membranes in 24-well plates. After 3 days for THP-1 cells and after 14 days for Saos-2 cells, the cells were fixed in 4% paraformaldehyde for 15 min, and the cells on materials in the inserts were frozen using Super Cryo Embedding Medium (SCEM, Section-Lab, Hiroshima, Japan). After removing the surrounding plastic of the inserts with a 6.0-mm biopsy punch, the specimens were further embedded in frozen medium in the provided stainless steel container. Then, 10-μm sections were cut by a cryomicrotome (Hyrax C60, Zeiss, Oberkochen, Germany) with an adhesive film (Cryofilm type 2C (16UF), Section-Lab) using a film method [16].

For immunocytochemistry (IHC), sections were permeabilized by treatment with PBS containing 0.2% Triton X-100. The sections were blocked in PBS containing 3% goat serum for 1 h. Subsequently, the sections were incubated overnight at 4 °C with mouse anti-human monoclonal antibodies (Santa Cruz Biotechnology, Dallas, TX, USA) against CD86 (dilution 1:50), CD206 (dilution 1:50) or osteocalcin (OCN, dilution 1:50) in blocking buffer. The bound antibodies were detected by incubation with horseradish peroxidase-conjugated secondary antibodies for 1 h. 3,3'-Diaminobenzidine was used to detect the expression of the investigated proteins. Counterstaining was performed with hematoxylin. Images of CD86 and CD206 IHC were captured with a digital microscope (VHX-6000, Keyence, Osaka, Japan), and OCN IHC images were captured with a light microscope (Nikon Eclipse E800, Nikon, Tokyo, Japan).

## Statistical analysis

For micro-CT analysis and cell viability assays, three and six independent specimens were assessed, respectively. The experiments for the real-time PCR assays were repeated 3 times in duplicate at each time point. The median and data ranges are presented, and data were analyzed for statistical significance using nonparametric Mann-Whitney tests (*p* values < 0.05 were considered significant) to compare the differences between the 2 granule sizes for each material by statistical software **(**GraphPad Prism 8.0 software, GraphPad Software Inc., La Jolla, CA, USA)**.**

## Results

### Characteristics of the tested BSs by micro-CT analysis

**Fig. 2:**
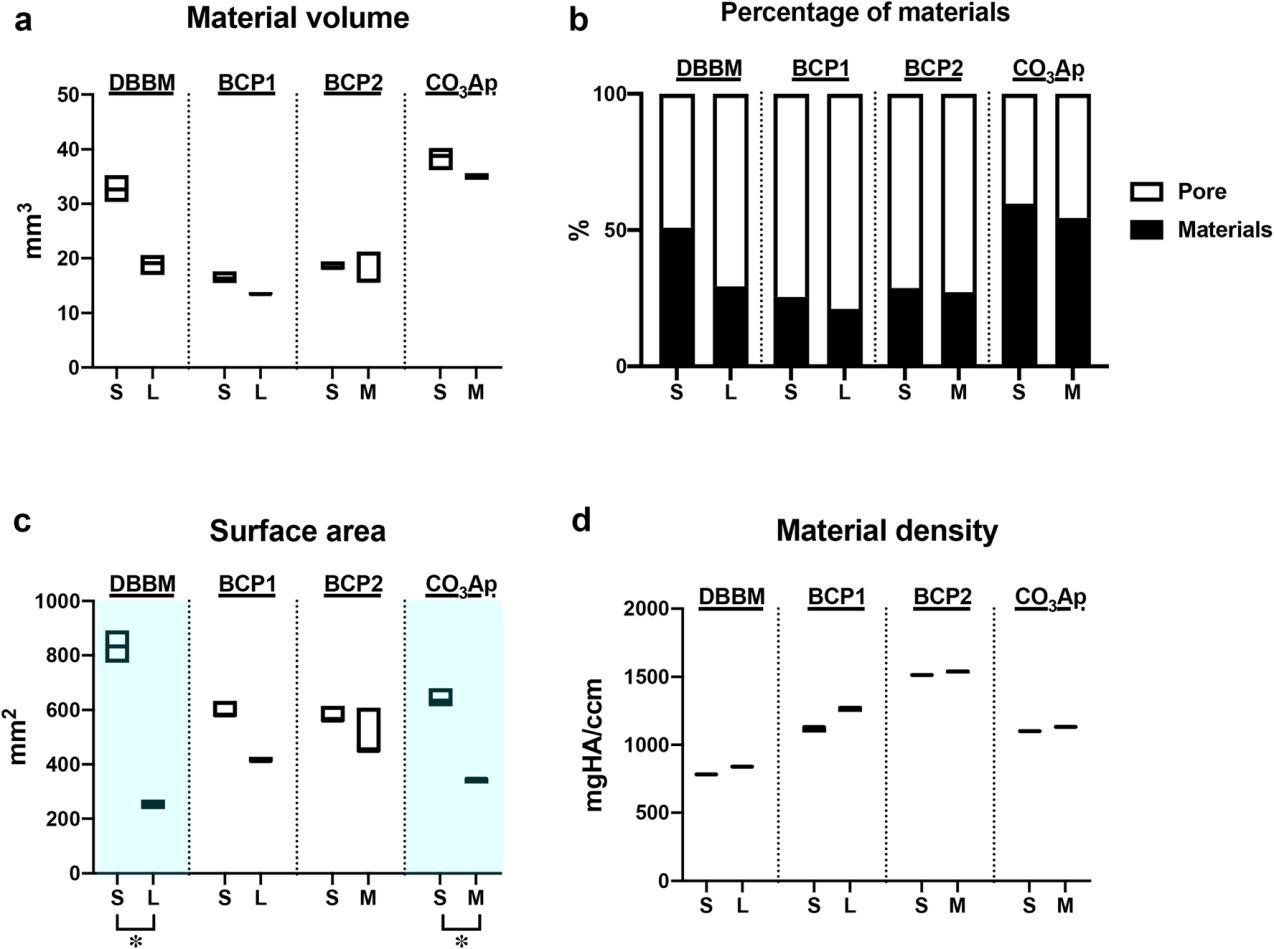
The quantified results of micro-CT analysis. (**a**) The material volume (mm^3^), (**b**) percentage of material filling (%), (**c**) surface area and (**d**) material density of BS granules in a 6.4 mm diameter × 2 mm height cylinder (* and light blue-colored areas denote significant differences between the groups, *p* < 0.05).

The two different granule sizes of the four BSs were first evaluated by micro-CT analysis to assess their characteristics (Figs. 1 and 2). The filling volume of BS in a cylinder of a standardized size (φ6.4 mm in diameter × 2.0 mm in height) depended on BS size in all BS groups (Fig. 1). In general, the quantitative analysis revealed that the smaller granules showed greater material volumes and surface areas than the larger granules due to their greater packing ability (Fig. 2a–c). Nevertheless, no significant differences were found in the material volume or percentage of materials in the cylinders between the two granule sizes for any BS (Fig. 2a, b). Interestingly, however, the quantitative results revealed that only DBBM and CO_3_Ap showed significantly greater surface area in smaller granules (S size) than in larger granules (L size for DBBM and M size for CO_3_Ap) (Fig. 2c). A similar material density was observed between the two granule sizes in all material groups, as expected (Fig. 2d).

### The effect of granule size on macrophage viability and polarization

**Fig. 3:**
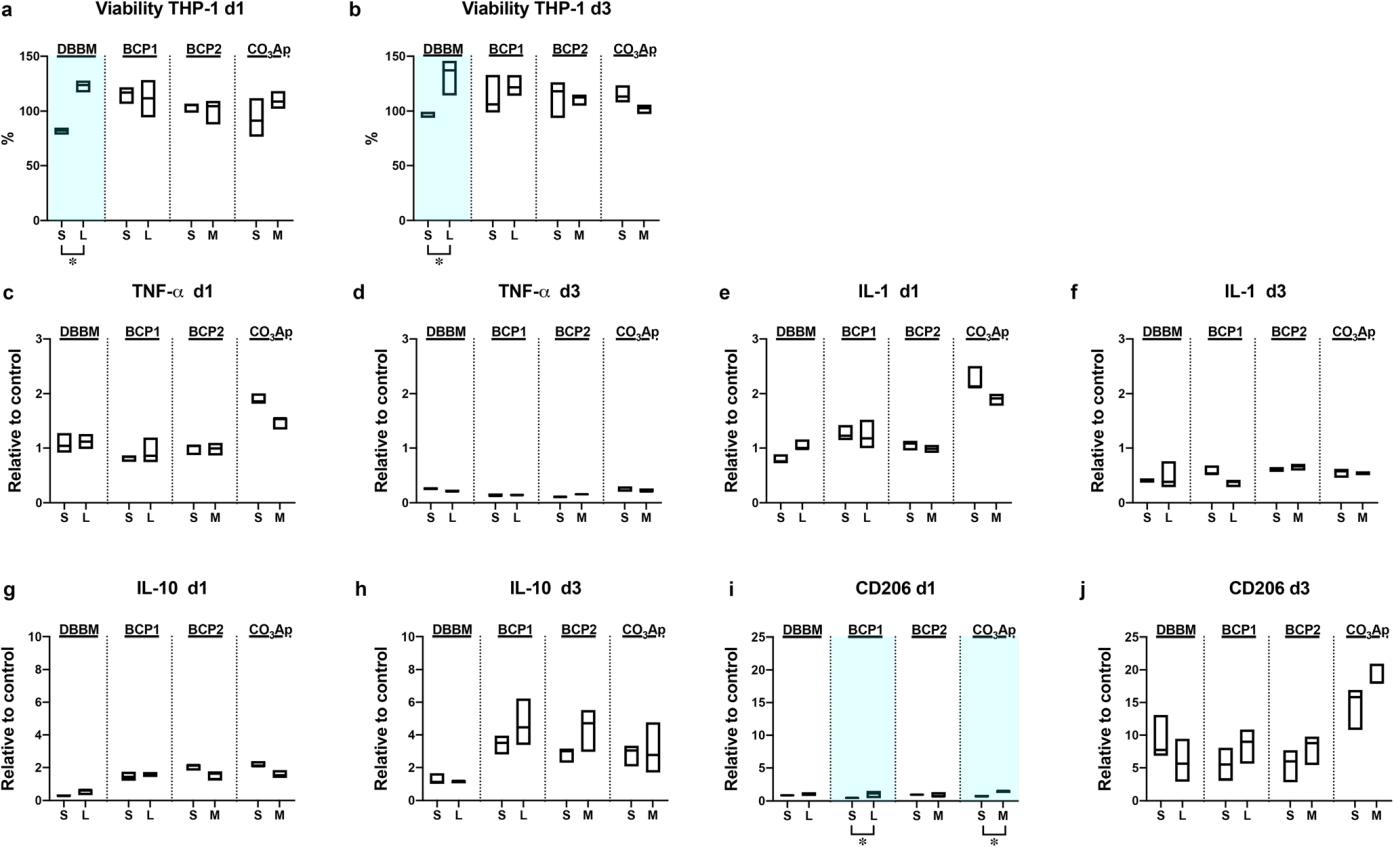
Macrophage behavior on the 2 sizes of BS granules. (**a**, **b**) Viability assay of THP-1-derived Mφs cultured on 2 sizes of granules on (**a**) day 1 and (**b**) day 3. (**c**–**j**) Macrophage polarization potential on the 2 sizes of BS granules. Real-time PCR of Mφs seeded on the granules was performed to assess the expression of the genes encoding (**c**, **d**) TNF-α, (**e**, **f**) IL-1, (**g**, **h**) IL-10, and (**i**, **j**) CD206 on day 1 (**c**, **e**, **g**, and **i**) and day 3 (**d**, **f**, **h**, and **j**) after cell seeding (* and light blue-colored areas denote significant differences between the groups, *p* < 0.05)

**Fig. 4:**
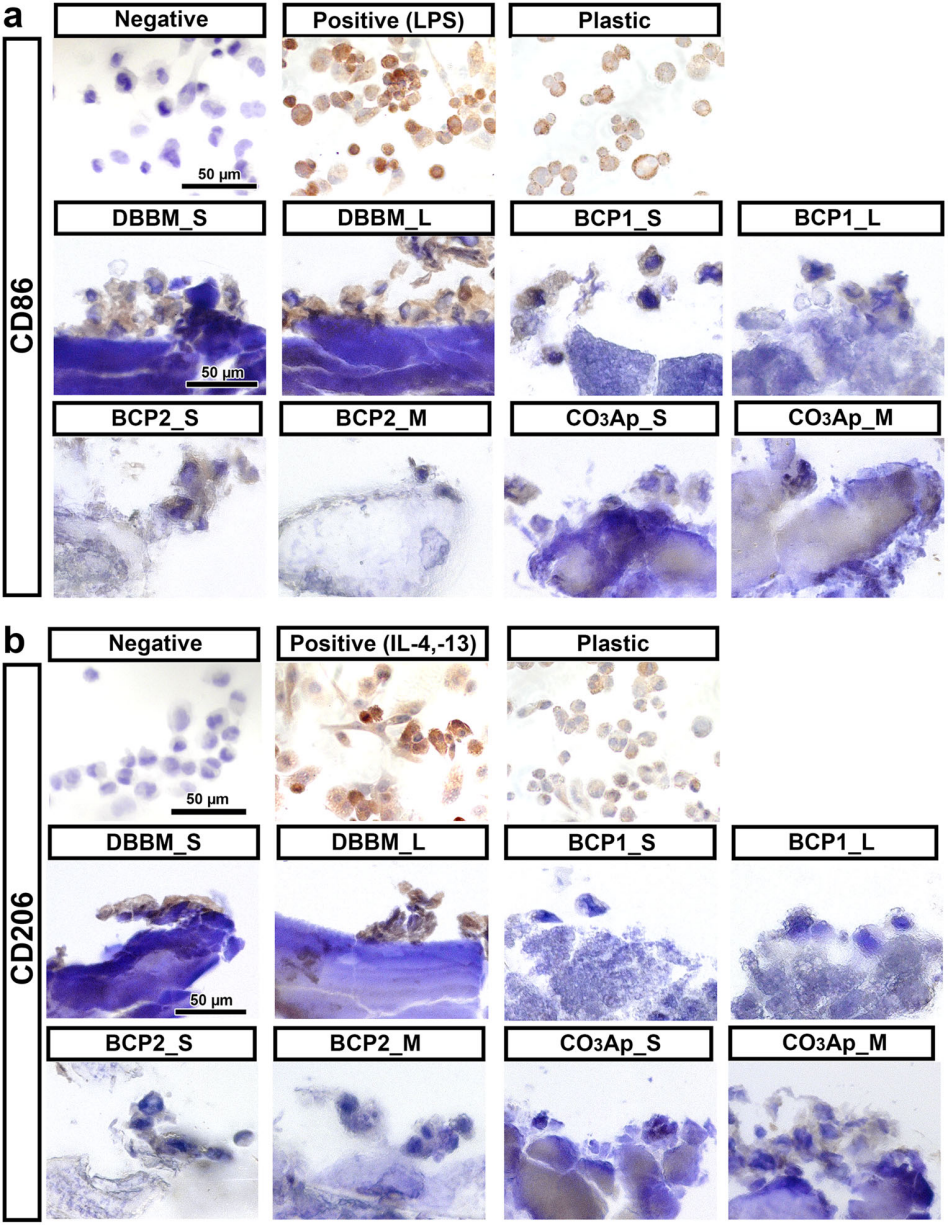
Immunochemical staining of (**a**) CD86 and (**b**) CD206 on undecalcified frozen sections of Mφs cultured on BS granules for 3 days

The viability and expression levels of genes involved in Mφ polarization were investigated by directly culturing THP-1-derived Mφs on the different BSs (Figs. 3 and 4). All the tested BSs showed excellent Mφ viability compared to the control (cell culture tissue plastic) on days 1 and 3 (Fig. 3a, b). Interestingly, higher viability was observed on days 1 and 3 in Mφs cultured on DBBM_L granules than in those cultured on DBBM_S granules (Fig. 3a, b). Nevertheless, the granule size of the other synthetic BSs (BCP1, BCP2, and CO_3_Ap) showed little effect on Mφ viability (Fig. 3a, b). Furthermore, the mRNA levels of Mφ polarization markers, including M1_Mφ markers (TNF-α and IL-1) and M2_Mφ markers (IL-10 and CD206), in response to each BS were investigated on days 1 and 3 (Fig. 3c–j). Interestingly, relatively higher mRNA levels of M1 markers (TNF-α and IL-1) on day 1 and an M2 marker (CD206) on day 3 were observed in cells on CO_3_Ap than in cells on the other BSs (Fig. 3c–j). However, few differences were observed in Mφ polarization marker expression between the 2 granule sizes in any of the BS groups (Fig. 3c–j). Only the mRNA levels of CD206 in the BCP1 and CO_3_Ap groups showed significant differences between the 2 granule sizes on day 1 (Fig. 3i). Immunochemical staining of Mφs on different BSs using the undecalcified frozen section technique showed that CD86 and CD206 were slightly expressed (intermediate expression levels between the positive and negative control values) in all BS groups on day 3. Nevertheless, no clear differences in expression patterns were found between the two granule sizes for any of the groups (Fig. 4).

### The effect of granule size on osteoblast viability and differentiation

**Fig. 5:**
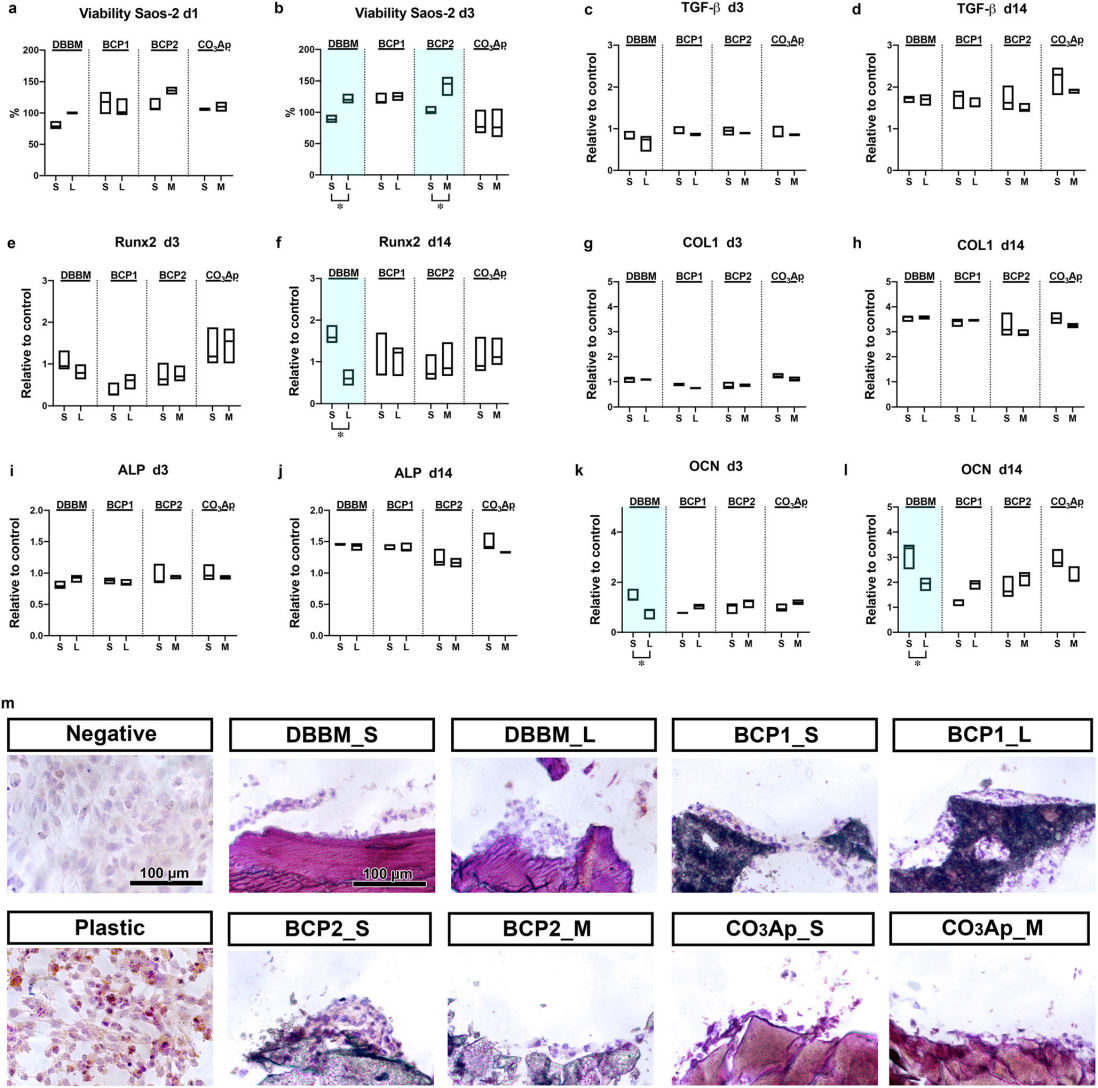
Osteoblast behavior on the 2 sizes of BS granules. (**a**, **b**) Viability assay of Saos-2 cells cultured on 2 sizes of BS granules on (**a**) day 1 and (**b**) day 3. (**c**–**l**) Real-time PCR of Saos-2 cells seeded on 2 BS granules was performed to assess the expression of the genes encoding (**c**, **d**) TGF-β, (**e**, **f**) Runx2, (**g**, **h**) COL1, (**i**, **j**) ALP, and (**k**, **l**) OCN at 3 days (**c**, **e**, **g**, **i**, and **k**) and 14 days (**d**, **f**, **h**, **j**, and **l**) after cell seeding. (m) Immunochemical staining of OCN on undecalcified frozen sections of Saos-2 cells cultured on BS granules for 14 days (* and light blue-colored areas denote significant differences between the groups, *p* < 0.05)002E

Saos-2 cells were cultured on the different BS granules and investigated for their viability and osteogenic potential (Fig. 5). As observed in the Mφs, all the tested BSs generally showed excellent Saos-2 cell viability compared to the control (cell culture tissue plastic) on days 1 and 3 (Fig. 5a, b). Significantly higher viability was observed in Saos-2 cells cultured on the larger DBBM and BCP2 (DBBM_L size and BCP2_M size) granules on day 3 than in those cultured on the smaller (DBBM_S size and BCP2_S size) granules (Fig. 5b). Real-time PCR experiments confirmed higher mRNA levels of growth factors and osteoblast differentiation markers, including TGF-β, COL1, ALP, and OCN, on day 14 in cells cultured on all BSs than in the control cells (Fig. 5c–l). However, similar trends in osteoblast differentiation potential were observed between the two granule sizes of each BS, although significantly higher mRNA levels were observed for Runx2 at 14 days and OCN at 3 and 14 days in cells cultured on the DBBM_S-size granules than in those cultured on the DBBM_L-size granules (Fig. 5c–l). Immunochemical staining of OCN in Saos-2 cells cultured on the tested BSs showed slightly positive expression in all BS groups (Fig. 5m). Once again, similar trends in OCN expression patterns were generally observed between the two granule sizes of each BS (Fig. 5m). However, in line with real-time PCR results, the DBBM_S-size granules resulted in higher OCN expression than the DBBM_L-size granules (Fig. 5m).

## Discussion

Four commercial BSs were investigated in the present study to determine the impacts of BS granule size on the regenerative potentials of THP-1-derived Mφs and osteoblast-like Saos-2 cells. The material surface area is an important factor, and the International Organization for Standardization (ISO) recommends using the standardized material surface area to assess biological responses to dental materials [17]. In the present study, however, the granules used were standardized throughout the experiments by utilizing the volume-based defect filling size concept instead of the weight-based concept. This approach was intended to increase the similarity of the experimental procedures to the clinical reality of the bone augmentation procedure [15]. The micro-CT imaging analysis revealed that the smaller granules showed higher material volumes and surface areas than the larger granules, as expected (Figs. 1 and 2). It was expected that the DBBM and CO_3_Ap granules, which showed significant differences in surface area between the S and M//L sizes, would further demonstrate different experimental outcomes in cells cultured on the two different sizes of BS.

Investigation of the response of immune cells to BS is used to screen the biocompatibility of biomaterials in vitro. Biomaterial-associated Mφs can switch between proinflammatory Mφs (M1_Mφs) and tissue regenerative Mφs (M2_Mφs) [13]. The biomaterial initially induces the M1 response to recruit inflammatory cells to the wound site and instigate the foreign body reaction, which is followed by a transition to the M2 phenotype; this transition is believed to be a favorable adaptation [18]. The osteopromotive capacities of BS implants are closely associated with the inflammatory response, as determined by biomaterial-associated Mφ polarization and functional status [19]. Therefore, the behaviors of Mφs and osteoblasts were investigated during the culture of cells on BSs in the present study.

Interestingly, the Mφ culture experiments on BSs showed a significantly higher viability of Mφs cultured on the DBBM_L-size granules at 1 and 3 days than of those cultured on the DBBM_S-size granules (Fig. 3). However, few differences were observed in the expression of Mφ polarization markers between the two granule sizes in any BS group, as assessed by real-time PCR and immunochemical staining (Figs. 3 and 4). DBBM granules have previously been shown to drive polarization toward the M2 phenotype, as indicated by high expression of CD163 and CD206 in the murine-derived macrophage cell line RAW 264.7 and by high expression of IL-10, Arginase 1 (Arg1), and CD206 in THP-1 cell-derived Mφs [15, 20]. BCP has been reported to upregulate the gene expression of most inflammatory factors and growth factors in RAW 264.7 cells [21, 22]. Nevertheless, Chen et al. reported that BCP increased the proportion of CD206-positive M2_Mφs, upregulated the expression of M2 markers in vitro, and increased the size of the Arg-positive M2 population in vivo. Furthermore, it was suggested that β-TCP promoted the secretion of M1 markers, whereas HAp resulted in a moderate number of both M1_Mφs and M2_Mφs [19]. The Mφ polarization trends on BCP might vary due to the ratio of resorbable β-TCP and nonresorbable HAp. To the best of the authors’ knowledge, the effects of CO_3_Ap granules on Mφ polarization have not yet been clearly reported. Interestingly, however, the real-time PCR experiment in the present study demonstrated that CO_3_Ap tended to change Mφ polarization; higher mRNA levels of M1 markers (TNF-α and IL-1) and an M2 marker (CD206) on day 1 were observed in cells cultured on CO_3_Ap than in cells cultured on the other BSs (Fig. 3). Nevertheless, a limitation of the present in vitro study was that we were unable to evaluate the switch between the M1 and M2 phenotypes caused by BSs.

Osteoblast culture experiments on BSs are commonly used to evaluate the in vitro cytotoxicity or cytocompatibility of BSs. The larger DBBM and BCP2 granules showed significantly increased viability on day 3 compared to that of the respective smaller granules (Fig. 5b). A similar trend for osteoblast differentiation was observed between the two different size groups (S size versus M/L sizes) in the Saos-2 cells (Fig. 5c–m). Nevertheless, once again, only DBBM induced significant differences in osteogenic marker levels, as illustrated by the higher mRNA levels of Runx2 at 14 days and of OCN at 3 and 14 days in the DBBM_S-size group compared to the DBBM_L size group (Fig. 5f, k, and l).

The preparation of undecalcified frozen sections and immunochemical staining were performed by Kawamoto’s film methods [16] and were used to evaluate differentiation marker expression at the protein level (Figs. 4 and 5m). This procedure was useful for immunostaining because it caused minimal damage to cell attachments to the BSs, and the immunostaining process was easier than the method used for decalcified paraffin sections [15]. Unfortunately, however, no noticeable differences were found at the protein level between the two sizes of each BS, except that the DBBM_S-size group resulted in higher OCN expression than the DBBM_L-size group (Fig. 5m).

One of the limitations of the present study may be the in vitro experimental model. For the cell experiments, the established human cell lines THP-1 and Saos-2 were selected due to the advantage of enhanced reproducibility of the results. However, it is impossible to perfectly mimic the real situation in the body in vitro regardless of the cell line selected. Furthermore, the comparison of data between two sizes of BS was the focus of the present study, and a nonparametric test was chosen due to the limited sample number. However, the limited sample number unfortunately did not allow us to perform correlation tests between BS material/size and biological parameters.

Notably, DBBM demonstrated clear differences between the S-size and L-size granules in material surface area, cell viability, and differentiation potential both in Mφs and osteoblasts. In line with the study using HAp by Dawson et al. [12], the present study suggests that the higher surface area of the DBBM_S-size granules might enlarge the cell adhesion area and promote differentiation more efficiently than that of the DBBM_L-size granules. Nevertheless, it was previously reported by Chackartchi et al. that there was no difference in clinical outcome between the two DBBM sizes (S size and L size) for sinus floor augmentation procedures at 6 to 9 months, as shown by both microcomputed tomography (CT) and histomorphometric analysis [23]. Thus, they suggested that the selection of DBBM granule size should depend on surgeon preference, sinus anatomy, or the presence of membrane rupture [23]. Testori et al., in contrast, showed results similar to those of the Chackartchi et al. study for DBBM_L-size granules (26.77% ± 9.63%) but had less favorable results for DBBM_S-size granules (18.77% ± 4.74%) [24]. It was also reported in an experimental animal study in minipigs by Jensen et al. that DBBM_S-size and DBBM_L-size granules performed equally well when used for sinus floor elevation with simultaneous implant placement with regard to the impact on the amount of newly formed bone, DBBM degradation, and bone-to-implant contact (BIC). Importantly, the DBBM_S-size granules showed significantly higher osteoconduction after 6 weeks (in the early period) than the DBBM_L-size granules [25]. The authors assumed that as the granule size decreases, the surface area increases; thus, the presentation of growth factors may be expected to increase correspondingly [25], as suggested by the present study.

Unfortunately, in vitro studies cannot perfectly reflect clinical outcomes; however, the present study is the first to investigate the impact of BS size on cell behavior using more than two commercial products. The differences in cell function between the two sizes of the tested commercial BSs must be small; however, the sensitivity of cells, including immune cells and osteoblasts, in response to different sizes of BSs might be relatively larger for DBBM than for other synthetic BSs, which might be associated with clinical outcome, especially osteoconductive potential, in particular cases. However, future preclinical and clinical studies are necessary to clarify the correlation between BS size and clinical outcomes for bone regenerative medicine.

## Conclusion

The effect of the size of BS on cell viability and differentiation was investigated in Mφs and osteoblasts in vitro. In the synthetic materials (BCP and CO_3_Ap), there were generally few effects of granule size on cell responses in both Mφs and osteoblasts. Nevertheless, in the present limited study, DBBM granules exhibited clear differences between the S size and L size in cell outcomes, including lower cell viability and a higher osteopromotive effect in the S-size group without noticeable changes to Mφ polarization. These data suggest that a smaller granule size might be advantageous due to an increase in bone regeneration potential, especially when using DBBM granules to treat defects.

## Supplementary information

Supplemental Table 1 (DOCX 14 kb).

## References

[1] Kao ST, Scott DD (2007). A review of bone substitutes. Oral Maxillofac Surg Clin North Am.

[2] Wessing B, Lettner S, Zechner W (2018). Guided bone regeneration with collagen membranes and particulate graft materials: a systematic review and meta-analysis. Int J Oral Maxillofac Implants.

[3] Darby I (2011). Periodontal materials. Aust Dent J.

[4] Zitzmann NU, Naef R, Schärer P (1997) Resorbable versus nonresorbable membranes in combination with Bio-Oss for guided bone regeneration. Int J Oral Maxillofac Implants 12:844–52 9425767

[5] Baldini N, De Sanctis M, Ferrari M (2011). Deproteinized bovine bone in periodontal and implant surgery. Dent Mater.

[6] Lobo SE, Livingston Arinzeh T (2010). Biphasic calcium phosphate ceramics for bone regeneration and tissue engineering applications. Materials.

[7] Driessens F (1980). The mineral in bone, dentin and tooth enamel. Bull Soc Chim Belg.

[8] Tas AC (2014). The use of physiological solutions or media in calcium phosphate synthesis and processing. Acta Biomater.

[9] Zapanta-LeGeros R (1965). Effect of carbonate on the lattice parameters of apatite. Nature.

[10] Ghanaati S, Barbeck M, Orth C, Willershausen I, Thimm BW, Hoffmann C, Rasic A, Sader RA, Unger RE, Peters F (2010). Influence of β-tricalcium phosphate granule size and morphology on tissue reaction in vivo. Acta Biomater.

[11] Tanuma Y, Anada T, Honda Y, Kawai T, Kamakura S, Echigo S, Suzuki O (2012). Granule size–dependent bone regenerative capacity of octacalcium phosphate in collagen matrix. Tissue Eng A.

[12] Dawson ER, Suzuki RK, Samano MA, Murphy MB (2018). Increased internal porosity and surface area of hydroxyapatite accelerates healing and compensates for low bone marrow mesenchymal stem cell concentrations in critically-sized bone defects. Appl Sci.

[13] Miron RJ, Bosshardt DD (2016). OsteoMacs: key players around bone biomaterials. Biomaterials.

[14] Cho SW, Soki FN, Koh AJ, Eber MR, Entezami P, Park SI, van Rooijen N, McCauley LK (2014). Osteal macrophages support physiologic skeletal remodeling and anabolic actions of parathyroid hormone in bone. Proc Natl Acad Sci U S A.

[15] Fujioka-Kobayashi M, Marjanowski SD, Kono M, Katagiri H, Miron RJ, Schaller B (2020) In vitro comparison of macrophage polarization and osteoblast differentiation potentials between granules and block forms of deproteinized bovine bone mineral. Materials (Basel) 13. 10.3390/ma13122682 10.3390/ma13122682 PMC7345324 32545502

[16] Kawamoto T, Kawamoto K (2014) Preparation of thin frozen sections from nonfixed and undecalcified hard tissues using Kawamot’s film method (2012). Methods Mol Biol 1130:149–164 10.1007/978-1-62703-989-5_11 24482171

[17] Iso I (2009). 10993–5: 2009 Biological evaluation of medical devices—part 5: tests for in vitro cytotoxicity.

[18] Sridharan R, Cameron AR, Kelly DJ, Kearney CJ, O’Brien FJ (2015). Biomaterial based modulation of macrophage polarization: a review and suggested design principles. Mater Today.

[19] Chen X, Wang M, Chen F, Wang J, Li X, Liang J, Fan Y, Xiao Y, Zhang X (2020). Correlations between macrophage polarization and osteoinduction of porous calcium phosphate ceramics. Acta Biomater.

[20] Shi M, Wang C, Wang Y, Tang C, Miron RJ, Zhang Y (2018). Deproteinized bovine bone matrix induces osteoblast differentiation via macrophage polarization. J Biomed Mater Res A.

[21] Wang J, Liu D, Guo B, Yang X, Chen X, Zhu X, Fan Y, Zhang X (2017). Role of biphasic calcium phosphate ceramic-mediated secretion of signaling molecules by macrophages in migration and osteoblastic differentiation of MSCs. Acta Biomater.

[22] Wang J, Su Y, Xu L, Li D (2020) Micro-patterned surface construction on BCP ceramics and the regulation on inflammation-involved osteogenic differentiation. Mater Sci Eng C Mater Biol Appl 116:111220 10.1016/j.msec.2020.111220 32806223

[23] Chackartchi T, Iezzi G, Goldstein M, Klinger A, Soskolne A, Piattelli A, Shapira L (2011). Sinus floor augmentation using large (1-2 mm) or small (0.25-1 mm) bovine bone mineral particles: a prospective, intra-individual controlled clinical, micro-computerized tomography and histomorphometric study. Clin Oral Implants Res.

[24] Testori T, Wallace SS, Trisi P, Capelli M, Zuffetti F, Del Fabbro M (2013) Effect of xenograft (ABBM) particle size on vital bone formation following maxillary sinus augmentation: a multicenter, randomized, controlled, clinical histomorphometric trial. Int J Periodontics Restorative Dent 33:467−75 10.11607/prd.1423 23820706

[25] Jensen SS, Aaboe M, Janner SF, Saulacic N, Bornstein MM, Bosshardt DD, Buser D (2015). Influence of particle size of deproteinized bovine bone mineral on new bone formation and implant stability after simultaneous sinus floor elevation: A histomorphometric study in minipigs. Clin Implant Dent Relat Res.

