# Supplementary material for: The impact of the size of bone substitute granules on macrophage and osteoblast behaviors in vitro
Source: Clin Oral Investig. 2021 Feb 4;25(8):4949–58. doi: 10.1007/s00784-021-03804-z (PMC8342374; doi:10.1007/s00784-021-03804-z)
Supplement: Supplementary file 1 — (DOCX 14 kb). [file 784_2021_3804_MOESM1_ESM.docx]

**Supplemental Table 1:** List of primer sequences for real-time PCR

| **Gene** | **Primer Sequence (5’ – 3’)** | |
| --- | --- | --- |
|  | **Forward** | **Reverse** |
| TNF-α | CAGCCTCTTCTCCTTCCTGAT | GCCAGAGGGCTGATTAGAGA |
| IL-1 | GGTTGAGTTTAAGCCAATCCA | TGCTGACCTAGGCTTGATGA |
| IL-10 | GAGGCTACGGCGCTGTCA | TCCACGGCCTTGCTCTTG |
| CD206 | GGGTTGCTATCACTCTCTATGC | TTTCTTGTCTGTTGCCGTAGTT |
| TGF-β | ACTACTACGCCAAGGAGGTCA | TGCTTGAACTTGTCATAGATTTCG |
| Runx2 | TCTTAGAACAAATTCTGCCCTTT | TGCTTTGGTCTTGAAATCACA |
| COL1 | CCCAGCCAAGAACTGGTATAGG | GGCTGCCAGCATTGATAGTTTC |
| ALP | GACCTCCTCGGAAGACACTC | TGAAGGGCTTCTTGTCTGTG |
| OCN | AGCAAAGGTGCAGCCTTTGT | GCGCCTGGGTCTCTTCACT |
| GAPDH | AGCCACATCGCTCAGACA | GCCCAATACGACCAAATCC |

TNF-α; Tumor necrosis factor-α, IL; interleukin, TGF-β1; Transforming growth factor-β, Runx2; Runt-related transcription factor 2, COL1; collagen 1, ALP; alkaline phosphatase, OCN; osteocalcin
